# Is dopamine transporter-mediated dopaminergic signaling in the retina a noninvasive biomarker for attention-deficit/ hyperactivity disorder? A study in a novel dopamine transporter variant Val559 transgenic mouse model

**DOI:** 10.1186/s11689-017-9215-8

**Published:** 2017-12-28

**Authors:** Heng Dai, Chad R. Jackson, Gwynne L. Davis, Randy D. Blakely, Douglas G. McMahon

**Affiliations:** 1 Department of Biological Sciences, Vanderbilt University, Box 35-1634 Station B, Nashville, TN 37235-1634 USA; 2 Department of Biomedical Sciences, Charles E. Schmidt College of Medicine and Brain Institute, Florida Atlantic University, Jupiter, FL 33458 USA; 3 Present address: Defense Threat Reduction Agency, 8211 Terminal Road, Lorton, VA 22079 USA

**Keywords:** Biomarker, Dopamine transporter, Electroretinogram, Attention-deficit/hyperactivity disorder, Ala559Val coding substitution, Anomalous dopamine efflux

## Abstract

**Background:**

Dopamine (DA) is a critical neuromodulator in the retina. Disruption of retinal DA synthesis and signaling significantly attenuates light-adapted, electroretinogram (ERG) responses, as well as contrast sensitivity and acuity. As these measures can be detected noninvasively, they may provide opportunities to detect disease processes linked to perturbed DA signaling. Recently, we identified a rare, functional DA transporter (DAT, SLC6A3) coding substitution, Ala559Val, in subjects with attention-deficit/hyperactivity disorder (ADHD), demonstrating that DAT Val559 imparts anomalous DA efflux (ADE) with attendant physiological, pharmacological, and behavioral phenotypes. To understand the broader impact of ADE on ADHD, noninvasive measures sensitive to DAT reversal are needed.

**Methods:**

Here, we explored this question through ERG-based analysis of retinal light responses, as well as HPLC measurements of retinal DA in DAT Val559 mice.

**Results:**

Male mice homozygous (HOM) for the DAT Val559 variant demonstrated increased, light-adapted ERG b-wave amplitudes compared to wild type (WT) and heterozygous (HET) mice, whereas dark-adapted responses were indistinguishable across genotypes. The elevated amplitude of the photopic light responses in HOM mice could be mimicked in WT mice by applying D_1_ and D_4_ DA receptor agonists and suppressed in HOM mice by introducing D_4_ antagonist, supporting elevated retinal DA signaling arising from ADE. Following the challenge with amphetamine, WT exhibited an increase in light-adapted response amplitudes, while HOM did not. Total retinal DA content was similar across genotypes. Interestingly, female DAT Val559 HOM animals revealed no significant difference in photopic ERG responses when compared with WT and HET littermates.

**Conclusions:**

These data reveal that noninvasive, in vivo evaluation of retinal responses to light can reveal physiological signatures of ADE, suggesting a possible approach to the segregation of neurobehavioral disorders based on the DAT-dependent control of DA signaling.

## Background

The neurotransmitter dopamine (DA) exerts powerful control over brain circuits that regulate reward, attention, and locomotor activity [1–3]. Accordingly, DA dysfunction is believed to contribute to the etiology of several neuropsychiatric disorders, including attention-deficit/hyperactivity disorder (ADHD) [4, 5], bipolar disorder (BPD) [6], schizophrenia [7, 8], and Parkinson’s disease [9–11]. Interestingly, increasing evidence supports findings that patients with these diseases exhibit impaired retinal and visual functions, suggesting that altered DA signaling in the retina may be under the control of the same molecular perturbations that support the etiology of these disorders [12] and that assessment of retinal DA signaling might offer a novel window into the diagnosis and treatment of neuropsychiatric disorders.

In the retina, DA mediates light adaptation and exhibits circadian rhythms of synthesis and release, such that DA signaling is higher during the daytime and during light exposure [13, 14]. DA is secreted by amacrine neurons in the inner nuclear layer of the retina, and it mediates feedback of photic information to the outer retina from the inner retina [15, 16]. DA-secreting amacrine cells influence other retinal neurons through volume conduction [17]. Among the retinal targets of DA, the influence of DA on electrical synapses is well described. Specifically, DA uncouples the gap junctions between horizontal cells [18], AII amacrine cells [19, 20], and rods and cones [21], leading to a reduction of receptive field size and blockade of rod signaling to ganglion cells. As a result, retinal circuits are reconfigured to a light-adapted state with increased light-induced response amplitudes in the presence of background light and enhanced acuity [12]. Retinal DA signaling is reflected in the amplitude of the photopic electroretinogram (ERG) with retinal-specific DA depletion producing decreased ERG amplitudes and rescue of retinal DA levels with L-DOPA restoring ERG amplitudes [12]. In addition, contrast sensitivity, spatial acuity, and circadian rhythms of light-adapted responses are all compromised in absence of retinal DA, further confirming that DA is important for light-adaptive mechanisms [12]. DA exerts its action on target neurons and circuits through D_1_-like (D_1_ and D_5_) and D_2_-like (D_2_, D_3_, and D_4_) receptors. In the retina, D_4_ receptor-medicated signaling pathways modulate light-adapted ERG rhythms and contrast sensitivity, whereas D_1_ receptor signaling contributes to high light-adapted ERG b-wave amplitudes and high spatial resolution [12].

The DA transporter (DAT, SLC6A3) is a key determinant of DA signaling capacity in the brain, limiting the action of the neurotransmitter through high-affinity clearance of extracellular DA, with recycling of DA into the presynaptic cytosol [22]. In the absence of DAT, extracellular DA levels are elevated in the striatum [23] whereas intra-neuronal levels of DA are decreased [22, 24]. The psychostimulant amphetamine (AMPH), structurally similar to DA, competes with extracellular DA at DAT and also induces DAT-mediated, non-vesicular release of cytosolic DA, providing two routes for elevation of extracellular DA levels. AMPH formulations and other agents that elevate extracellular DA (e.g., methylphenidate, MPH, Ritalin™) are also commonly prescribed for the treatment of ADHD (e.g., Adderall [25, 26]). In addition to its significant expression in the brain [27], DAT is also expressed in the somata and processes of dopaminergic amacrine cells in rat and mouse retina [28, 29]. In DAT knockout mice, a significant decrease in retinal sensitivity is observed under dark-adapted (scotopic) conditions [30]. DAT has also been suggested to play a role in form-deprivation myopia, as DAT binding in myopic retinas is lower than that in the normal control eyes [31].

Genetic variation in DAT has functional consequences for brain DA signaling and behavior. Recently, we identified a rare human DAT coding substitution (DAT Ala559Val) in two male siblings diagnosed with ADHD [5]. The Val559 variant had been previously identified in a female subject with bipolar disorder (BPD) [32] and following our ADHD report, was identified in two unrelated male subjects with autism spectrum disorder (ASD) [33]. In both heterologous expression studies [34, 35] and in the DAT Val559 knock-in mouse model [34, 36], there was anomalous dopamine efflux (ADE) consistent with changes in DAT function. In the mouse model, we observed an altered pattern of locomotion with decreased vertical activity and increased horizontal locomotion speed (darting) in response to imminent handling, significantly elevated extracellular levels of striatal DA under basal conditions without a change in DA tissue content, and a blunted response to AMPH or MPH paralleled by reduced locomotor activation by these psychostimulants [36]. We have previously proposed [34] that ADHD drugs containing AMPH formulation block the ADE of DAT Val559, which is distinct from blocking reuptake. In the former case, normal excitation coupling to vesicular release is restored, whereas in the latter case, the coupling to release is not modulated, only the amplitude of the response. We propose that it is the “noise” from leak that is more of a problem, at least for a subset of subjects, and thus, release of DA cannot be sensed appropriately. Ex vivo brain slice studies revealed tonic presynaptic DA receptor activation that supported a blunting of depolarization-evoked DA release. Altogether, our findings in the DAT Val559 model reveal a state of tonic hyperdopaminergia that leads to changes in locomotor patterns and anomalous responses to psychostimulants.

Although rare, the DAT Val559 variant may represent a genetic form of a functional state common to the broader etiology of idiopathic ADHD. If true, non-invasive tests of DA action that can be employed in ADHD subjects demonstrating ADE may allow for improved ADHD diagnosis and/or treatment. In the current study, we sought to evaluate DA-sensitive measures in the retina that can be detected using ERG. Specifically, we determined whether the DAT Val559 allele alters light-adapted retinal responses under basal conditions and as a consequence of AMPH administration. We observed DAT Val559 animals exhibit retinal responses consistent with the reported role of the variant in elevating tonic dopaminergic signaling and blunted responses to AMPH. Moreover, we observed differential retinal responses dependent on sex, of interest given the sex bias in ADHD diagnoses [37].

## Methods

### Animal usage and care

All animal protocols were approved and in accordance with the guidelines established by the Vanderbilt University Animal Care Division and the National Institutes of Health Guide for the Care and Use of Laboratory Animals. WT, homozygous (HOM), and heterozygous (HET) Val559 DAT littermates with a hybrid background (∼ 75% 129S6/SvEvTac and ∼ 25% C57BL/6 J) [36] were reared in a 12-h-light and 12-h-dark lighting condition. Only animals aged postnatal day 40 (P40) to P120 were subject to further tests. Unless otherwise noted, mice were tested or humanely killed during the middle of light phase of their light cycles (10:00 A.M.–2:00 P.M, Central Standard Time). The light intensity of the housing room was 100 ± 15 lx, provided by fluorescent bulbs. Mice were provided with water and food ad libitum.

### ERG

The ERG was used to measure global retinal responses to light stimuli using the LKC Technologies UTAS visual electrodiagnostic test system (Gaithersburg, MD). Scotopic and photopic ERG recordings were performed as previously described [12, 38]. All animals were dark-adapted overnight (~ 16–20 h) and tested during 4–8 h after subjective light onset (6:00 A.M., Central Standard Time). Mice were anesthetized with an intraperitoneal injection (IP injection) of ketamine (70 mg/kg) and xylazine (7 mg/kg), and their pupils dilated with 1% tropicamide (AKORN, NDC17478–102-12, Lake Forest, IL) under dim red light (Kodak GBX-2 Safelight, Rochester, NY). Their eyes were kept moist with 1% carboxymethyl-cellulose sodium eye drops (CVS, Extra Strength Lubricant Gel Drops Dry Eye Relief, Woonsocket, RI), and core body temperature was maintained at ∼ 37.0 °C using a thermostatically controlled heating pad regulated by a rectal temperature feedback probe (CWE, Model TC-1000 Temperature Controller, Ardmore, PA). Needle electrodes placed in the middle of forehead and the base of the tail served as reference and ground leads, respectively. A gold contact lens electrode was used for recording ERG responses (LKC Technologies; #N30).

Scotopic ERG responses were differentially amplified and filtered (bandwidth 0.3–500 Hz), with responses digitized at 1024 Hz. The recording epoch was 250 ms, with a 20-ms prestimulation baseline. Stimulus flashes were presented in an LKC BigShot ganzfeld. A total of 15 stimulus intensities, ranging from − 6.50 to 2.00 log cd*s/m^2^, were used under dark-adapted conditions. Each flash duration was 20 μs, and stimuli were presented in order of increasing intensity. As flash intensity increased, retinal dark adaptation was maintained by increasing the interstimulus interval from 30 to 180 s.

For photopic ERGs, mice were first given two flashes (−0.1 log cd*s/m^2^) under dark-adapted conditions to assess for a normal retinal response. A steady background-adapting field (40 cd/m^2^) inside the UTAS BigShot ganzfeld followed to saturate rod photoreceptors, and simultaneously, 0.90 log cd*s/m^2^ bright light flashes were presented at 0.75 Hz for a light adaptation session of 16 min. Data were collected and averaged in 2-min bins, totaling 8 bins. All other test parameters were similar to the scotopic ERG.

For the photopic ERG rescue experiment, IP injection of 1 mg/kg D_1_ receptor agonist (SKF38393, Sigma-Aldrich, Cat# D047, St. Louis, MO) and 1 mg/kg D_4_ receptor agonist (PD168077, Tocris Bioscience, Cat# 1065, Bristol, United Kingdom) were administered to WT mice 1 h before testing. Mice were injected under dim red light and returned to dark box until testing.

For the photopic ERG suppression experiment, IP injection of 1 mg/kg selective D_4_ DA receptor antagonist (L-745,870, Tocris Bioscience, Cat# 1002) was administered to HOM mice for 5 days 30 min prior to light onset in the morning. On the fifth day, animals were subject to photopic ERG tests.

The effects of D-AMPH on light-adapted ERG were explored by IP injections of 4 mg/kg AMPH to HOM and WT mice 15 min before the testing.

The scotopic a-wave was measured from the onset of flashes to the trough of the first negative deflection and b-wave was from the trough of the a-wave to the peak of the b-wave amplitude. Regarding photopic recordings, only b-wave amplitude could be reliably measured, which was defined as the difference from onset of stimuli to the peak of the b-wave.

### HPLC determination of DA and its metabolites

Animals from all groups were dark-adapted overnight (~ 16-20 h) and then sacrificed under either dark or light conditions. Retinas were collected, immediately frozen in liquid nitrogen in 1.5-mL tubes, and stored at − 80 °C until processed for HPLC analysis. Under dark conditions, mouse retinas were dissected from the whole eye and separated from the retinal pigment epithelium in the presence of dim red light (Kodak GBX-2 Safelight). Under light conditions, after approximately 15 min lighting exposure, retinas were obtained in the presence of room lighting similar to the background light during the photopic ERG test. HPLC analyses were conducted in the Vanderbilt Brain Institute Neurochemistry Core.

Retinas were homogenized, using a tissue dismembrator, in 100–750 μL of 0.1 M TCA, which contained 10^−2^ M sodium acetate, 10^−4^ M EDTA, 5 ng/mL isoproterenol (as internal standard), and 10.5% methanol (pH 3.8). Samples were spun in a microcentrifuge at 10,000*g* for 20 min. The supernatant was removed and stored at − 80 °C [39]. The pellet was saved for protein analysis. Supernatant was thawed and spun for 20 min, and samples of the supernatant were then analyzed for biogenic monoamines. Retinal biogenic amines were determined by HPLC using an Antec Decade II (oxidation 0.65) electrochemical detector operated at 33 °C. Twenty microliter samples of the supernatant were injected using a Water 2707 autosampler onto a Phenomenex Kinetex (2.6μm, 100Å) C18 HPLC column (100 × 4.60 mm). Biogenic amines were eluted with a mobile phase consisting of 89.5% 0.1 M TCA, 10^−2^ M sodium acetate, 10^−4^ M EDTA, and 10.5% methanol (pH 3.8). Solvent was delivered at 0.6 mL/min using a Waters 515 HPLC pump. Using this HPLC solvent, the following biogenic amines elute in the following order: dihydroxyphenylacetic acid (DOPAC), DA and homovanillic acid (HVA) [40, 41]. HPLC control and data acquisition are managed by Empower software. In this report, retinal biogenic amine analyses are represented as ng/mg protein. Total retinal protein concentration was determined using BCA Protein Assay Kit (Thermo Scientific, Cat# 23225, Waltham, MA). Ten-microliter tissue homogenate was distributed into a 96-well plate and 200 μL of mixed BCA reagent (25 mL of protein reagent A is mixed with 500 μL of protein reagent B) was added. Incubate the plate at room temperature for 2 h for the color development. A bovine serum albumin standard curve was generated at the same time, spanning the concentration range of 20–2000 μg/mL. Absorbance of standards and samples were measured at 562 nm. The inter-day variation of biogenic amine analysis using HPLC with electrochemical detection has been determined for the following analytes as: DOPAC, 2.3%; DA, 1.2%; 5-HIAA, 4.3%; HVA, 2.6%; 5-HT, 8.6%; and 3-MT, 10.2%. The intra-day variation for these analytes are DOPAC, 2.7%; DA, 0.8%; 5-HIAA, 1.2%; HVA, 2.6%; 5-HT, 8.8%; and 3-MT, 7.1%.

### Statistical analysis

Two-tailed *t* test and one- and two-way ANOVAs were used where applicable and reported. Post hoc analyses followed ANOVAs to confirm the difference among groups. Significance levels were set at *P* < 0.05 and represented as means ± SEM as indicated in each graph (Graphpad, La Jolla, CA and Sigmaplot, San Jose, CA).

## Results

### DAT Val559 homozygous male mice have increased light-adapted retinal responses

To evaluate the impact of the DAT Val559 variant on retinal function in vivo, we measured retinal responses in male DAT Val559 homozygous (HOM), heterozygous (HET), and wild type (WT) animals under dark-adapted and light-adapted conditions and used the ERG positive b-wave amplitude as the readout. All recordings were made at midday in the 12:12 light-dark cycle that the animals were maintained on, under dark-adapted or light-adapted conditions.

**Fig. 1:**
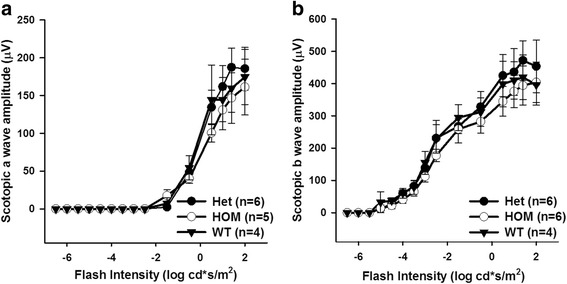
The dark-adapted ERG is not affected by the DAT Val559 mutation in male mice. Dark-adapted a-wave (**a**) and b-wave (**b**) amplitudes (μV) plotted as a function of stimulus intensity (log cd*s/m^2^) in Val559 homozygous (HOM: open circles), heterozygous (HET: filled circles), and wild type (WT: filled triangles) animals. There is no significant effect of genotype on either a-wave or b-wave as revealed by two-way ANOVA (a-wave: *F*_(2,180)_ = 1.146, *P* = 0.32; b-wave *F*_(2,195)_ = 2.530, *P* = 0.08; *n* = 4–6 mice). All data are represented as means ± SEM

Dark-adapted ERG responses were recorded after overnight dark adaptation. Full-field light flashes of increasing intensity were presented to the animals on a completely dark background. This test measures the summed responses of rod and cone photoreceptors (corneal negative a-wave) and ON-bipolar cell responses (corneal positive b-wave) to flashes of increasing light intensity. Although HOM showed a small decreasing trend in rod sensitivity, statistically, we observed no significant difference in a- and b-wave amplitudes in DAT Val559 HOM and HET animals compared to WT littermates under dark-adapted conditions (Fig. 1a, a-wave; *P* = 0.32; b, b-wave; *P* = 0.08, two-way ANOVA), in agreement with previous results with genetic suppression of retinal DA synthesis [12, 42].

**Fig. 2:**
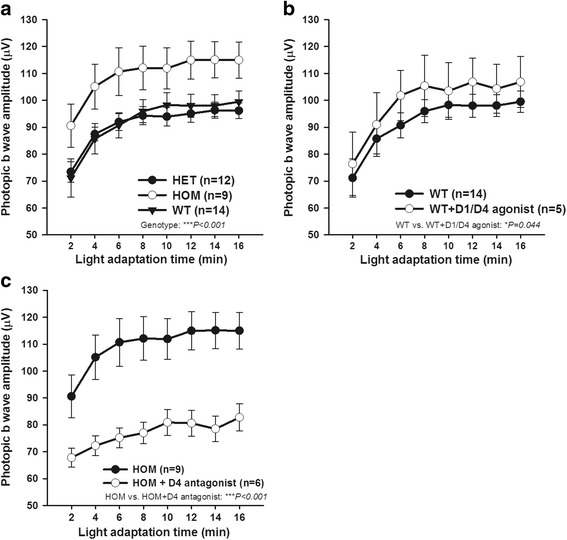
The DAT Val559 homozygous mutation affects light-adapted retinal function via dopaminergic signaling in male mice. **a**, **b**, and **c** Light-adapted (photopic) ERG b-wave amplitudes (μV) plotted as a function of light adaption time (minutes) in all groups. **a** Mice carrying homozygous Val559 mutation of DAT (HOM: open circles) have significantly higher photopic b-wave amplitudes compared to WT (filled triangles) and HET (filled circles; *F*_(2,256)_ = 26.98, ***P < 0.001, two-way ANOVA, *n* = 9–14 mice). **b** Injection of D_1_/D_4_ receptor agonists (SKF38393 and PD168077, 1 mg/kg, respectively) elevated the photopic ERG in WT animals (open circles) compared with untreated group (filled circles; *F*_(1,136)_ = 4.124, *P = 0.044, two-way ANOVA, *n* = 5–14 mice). **c** Injection of D_4_ receptor-selective antagonist L-745,870 (1 mg/kg) significantly reduced the response amplitude of DAT HOM (open circles; *F*_(1,104)_ = 82.06, ***P < 0.001, two-way ANOVA, *n* = 6–9 mice). All data are represented as means ± SEM

Light-adapted ERG responses were assessed using bright light flashes in the presence of rod-saturating, light-adapting background illumination over a period of approximately 16 min. This test assays the transition from dark-adapted to light-adapted vision, indicating the retina’s ability to adapt to background illumination. Cone-driven ERG responses increase over time as the retina adapts to the rod-saturating background, and this adaption is dependent on retinal DA [12]. Thus, we expected that mice harboring DAT Val559 would exhibit enhanced retinal ERG responses during light-adaptation. WT, DAT Val559 HOM, and HET groups all exhibited increases in b-wave amplitude with time in light adaptation. However, the DAT Val559 HOM mice displayed significantly elevated photopic b-wave amplitudes at each level of light adaptation as compared with WT and HET littermates, whereas WT and HET were indistinguishable (Fig. 2a; HOM vs. WT and HET, ***P < 0.001, two-way ANOVA). As the increased light-adapted ERG amplitude is mediated by DA acting through D_1_ and D_4_ receptors [12], we tested whether the increased b-waves observed in the DAT Val559 HOM mice also used these signaling pathways. The light-induced increase in b-wave amplitude in HOM was mimicked by applying D_1_ and D_4_ DA receptor agonists (SKF38393 and PD168077, 1 mg/kg, respectively) to dark-adapted WT mice (Fig. 2b; *P = 0.044, two-way ANOVA) and suppressed in HOM mice by the D_4_ antagonist (L-745,870, 1 mg/kg, 5-day injection) (Fig. 2c; ***P < 0.001, two-way ANOVA), suggesting altered retinal DA signaling due to the constitutive ADE of DAT Val559.

### Retinal responses to AMPH are blunted in male DAT Val559 HOM mice

**Fig. 3:**
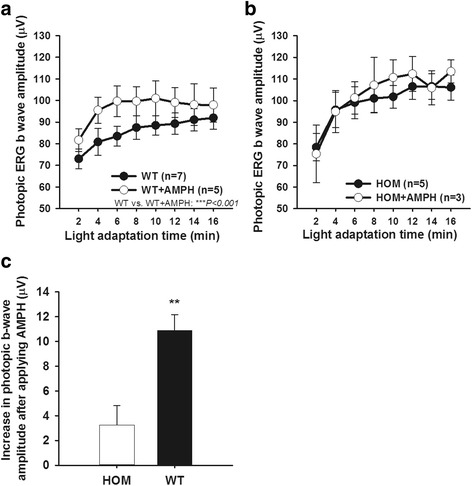
AMPH treatment alters photopic b-wave amplitudes differently in WT vs. DAT Val559 HOM in male mice. AMPH-treated (4 mg/kg) WT (**a**) (*F*_(1,80)_ = 12.758, ***P < 0.001, two-way ANOVA, *n* = 5–7 mice) showed increased light-adapted b-wave amplitudes, but homozygous mutants (**b**) were not affected by AMPH (*F*_(1,48)_ = 0.688, *P* = 0.411, two-way ANOVA, *n* = 3–5 mice). **c** The increments of ERG amplitude following AMPH injection were quantified by each genotype (HOM: white bar; WT: black bar) (*t* = −3.774, **P = 0.002, *t* test). All data are represented as means ± SEM

After observing a significant difference in light response amplitude between HOM and WT, we further challenged this mouse line with AMPH. DAT Val559 HOM knock-in mice show blunted AMPH-induced striatal DA elevations and motor activity in vivo [36]. To assess whether these alterations are penetrant at the level of the retina, AMPH was injected systemically followed by light-adapted ERG measurements. AMPH indeed increased the b-wave amplitudes in WT (Fig. 3a; ***P < 0.001, two-way ANOVA), but these effects were blunted in the retinas of HOM mice (Fig. 3b; *P* = 0.411, two-way ANOVA). The average increase of ERG amplitudes at each time point following AMPH treatment were quantified by genotypes (Fig. 3c; **P = 0.002, *t* test), in which WT displayed significant overall elevation of b-wave amplitude by AMPH compared to HOM, indicating a possible ceiling effect due to partial depletion of DA reservoir caused by ADE in HOM animals.

### Overall retinal DA content is not influenced by DAT Val559

**Fig. 4:**
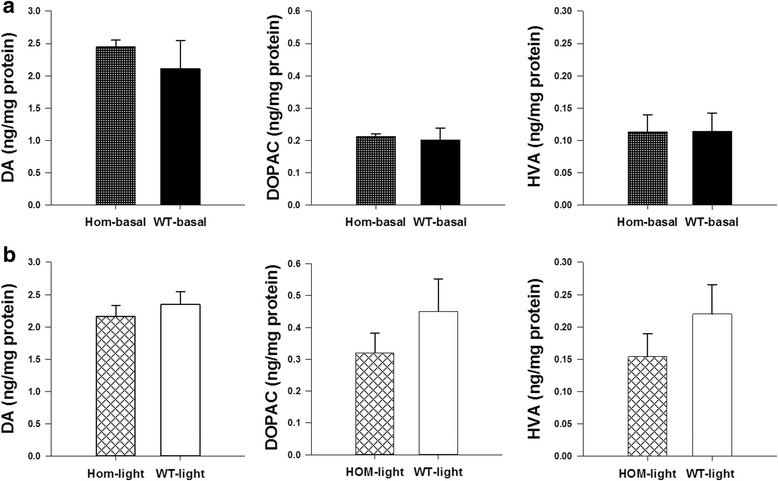
Retinal dopamine content and its metabolite levels do not differ in male DAT Val559 homozygous mutant mice. Retinal DA, DOPAC, and HVA were measured by HPLC either under complete darkness (**a** HOM: checked black bar, WT: black bar) or light conditions following 15–20 min of light exposure (**b** HOM: checked white bar, WT: white bar). Dopamine levels did not exhibit differences between genotypes or light conditions (HOM vs. WT, *F*_(1,23)_ = 0.554, *P* = 0.464; basal vs. light, *F*_(1,23)_ = 0.0605, *P* = 0.808, two-way ANOVA). HOM and WT also showed similar levels of DOPAC and HVA. Exposure to light only elevated the DOPAC and HVA levels in WT animals, whereas in HOM, the levels of these two metabolites remained statistically unchanged. (DOPAC: HOM vs. WT, *F*_(1,21)_ = 0.391, *P* = 0.538; basal vs. light, *F*_(1,21)_ = 4.364, *P = 0.049, two-way ANOVA; within WT, basal vs. light, *P < 0.05, post hoc Student-Newman-Keuls method) (HVA: HOM vs. WT, *F*_(1,23)_ = 0.531, *P* = 0.474; basal vs. light, *F*_(1,23)_ = 5.638, *P = 0.026, two-way ANOVA; within WT, basal vs. light, *P < 0.05, post hoc Student-Newman-Keuls method). Data are represented as means ± SEM (*n* = 6–8 mice)

The increased photopic ERG amplitudes in DAT Val559 HOM and pharmacological evidence suggested possible changes in retinal DA and its turnover, so we next measured total levels of DA and its metabolites with HPLC. However, the ADE of DAT Val559 mice has been found to result in no significant changes in tissue DA levels in the brain [36]. Similar to brain findings, HOM mice showed no significant genotype-dependent changes in basal retinal DA, DOPAC, and HVA levels (Fig. 4a) or after 20 min of light exposure (Fig. 4b; DA: *P* = 0.464; DOPAC: *P* = 0.538; HVA: *P* = 0.474, two-way ANOVA). This lack of increase in total DA content is also consistent with findings in the retina of DAT-knockout mice [30]. Retinal levels of DOPAC and HVA increased in WT mice in response to light, with an increase of 0.246 ng/mg for DOPAC (basal vs. light, *P < 0.05, two-way ANOVA, post hoc Student-Newman-Keuls method) and 0.124 ng/mg for HVA (basal vs. light, *P < 0.05, two-way ANOVA, post hoc Student-Newman-Keuls method). HOM mice, however, did not show different levels of DOPAC and HVA after 20 min of light exposure (DOPAC and HVA, basal vs. light, *P* > 0.05, two-way ANOVA, post hoc Student-Newman-Keuls method).

### The effect of DAT Val559 on photopic ERG amplitude is sex-dependent

**Fig. 5:**
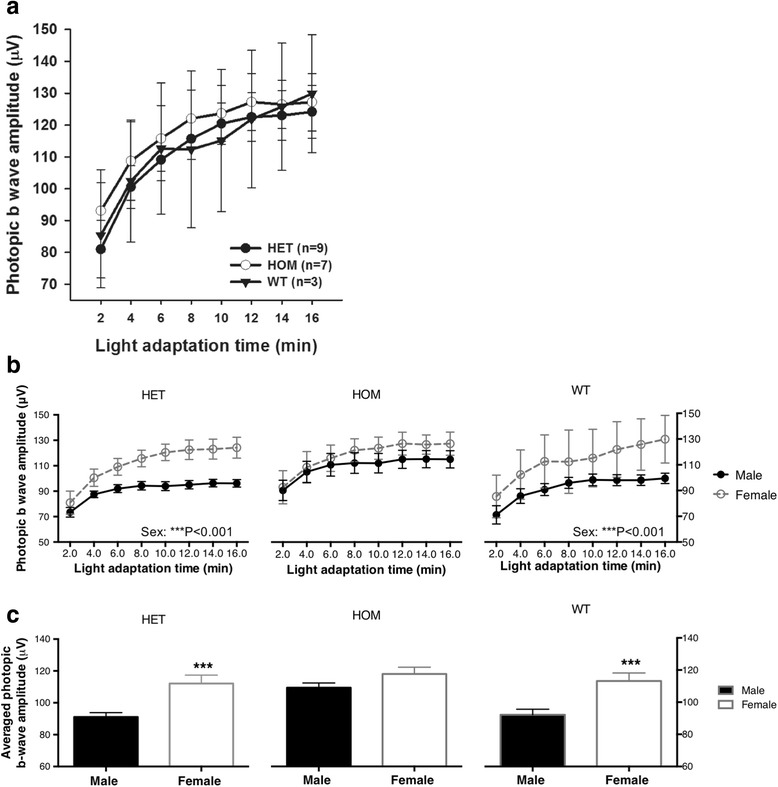
Female mice do not exhibit the effect of DAT Val559 on photopic ERG responses. **a** Photopic ERG b-wave amplitudes (μV) of female mice plotted as a function of light adaption time (minutes; HOM: open circles; HET: filled circles; and WT: filled triangles). The HOM group does not differ from HET or WT (*F*_(2,128)_ = 0.869, *P* = 0.422, two-way ANOVA, *n* = 3–9 mice). **b** Female HET (left column, open circles, *F*_(1,152)_ = 64.43, ***P < 0.001, two-way ANOVA, *n* = 9–12 mice) and WT (right column, open circles, *F*_(1,120)_ = 18.89, ***P < 0.001, two-way ANOVA, *n* = 3–14 mice) show elevated photopic amplitudes compared to the male cohort (filled circles), while amplitudes in HOM remained the same independent of sex (middle column, *F*_(1,112)_ = 3.860, *P* = 0.0519, two-way ANOVA, *n* = 7–9 mice). **c** Averaged increases were quantified by genotype and sex (from left to right, HET: **P = 0.0070, Mann-Whitney test; HOM: *P* = 0.052, Mann-Whitney test; WT: **P = 0.0038, t-test). All data are represented as means ± SEM

Because diagnosis of ADHD is more common in males, we initially focused our studies on male mice. Interestingly, upon testing a female cohort, we observed differential retinal light-adapted responses and effects of the DAT Val559 allele. Thus, in contrast to our findings in males, female DAT Val559 HOM animals revealed no significant difference in photopic ERG responses when compared with WT and HET littermates (Fig. 5a; *P* = 0.422, two-way ANOVA). The lack of difference was mainly due to increases in photopic b-wave amplitudes of female WT and HET in a comparison with male cohorts, which averaged 23.0% (Fig. 5b, c, left column, HET; ***P < 0.001, two-way ANOVA) and 22.8% (Fig. 5b, c, right column, WT; ***P < 0.001, two-way ANOVA), respectively. The light-adapted responses of DAT in female Val559 HOM animals were similar to the male HOM cohort in amplitude (Fig. 5b, c, middle column, HOM; *P* = 0.052, two-way ANOVA).

## Discussion

To evaluate the possible utility of retinal measures as an aid in evaluation of DA-linked neurobehavioral disorders and to further our understanding of the role of DA and DAT in the retinal function, we employed a mouse model that expresses the human DAT coding variant Val559. This variant has been demonstrated to disrupt multiple aspects of DA homeostasis and signaling in the brain in vivo, producing elevated basal striatal extracellular DA levels and blunted DA elevation upon local and systemic AMPH application [36]. Here, using noninvasive electrophysiological approaches, we assessed the effects of the variant on retinal DA and retinal function in intact mice and found that Val559 specifically enhanced the light-adapted ERG response amplitude and blunted AMPH enhancement of light response amplitude. We also found that the alteration of ERG amplitudes by the Val559 variant of DAT to be sex-dependent, with genotype effects detectible only in males.

### Val559 DAT increases light-adapted retinal responses

Retinal circuits are modulated dynamically according to background illumination, with rod and cone systems for dark-adapted and light-adapted vision, respectively. The reconfiguration of circuits for high-resolution light-adapted vision is achieved, in part, through intra-retinal retrograde signaling mechanisms mediated by retinal DA. The retina’s ability to adapt to a change in background illumination can be observed in the increased light-adapted ERG b-wave response mediated by light-stimulated release of DA [12]. Retinal DA also contributes to enhanced contrast sensitivity and high spatial resolution [12], through acting on gap junctions and chemical synaptic transmission, voltage-gated ion channels, and cAMP metabolism [43]. In mice in which tyrosine hydroxylase (TH, the rate-limiting enzyme of DA synthesis) is specifically depleted in the retina, retinal DA levels are markedly reduced, resulting in specific loss of the light-adapted ERG b-wave response amplitude [12]; however, brain DA levels remain normal. The deficiency in retinal DA is rescued by L-DOPA treatment and D_4_ receptor agonist, indicating the indispensable role of retinal DA in regulating photopic ERG responses.

In addition to the synthesis pathway, DA signaling is tightly coordinated through DAT-mediated DA clearance. Our results demonstrate that male mice homozygous for the Val559 DAT allele exhibit increased photopic b-wave amplitudes compared to WT and heterozygous littermates. The suppression of these elevated responses by D_4_ receptor antagonist in Val559 HOM and the increase in these responses by D_1_/D_4_ receptor agonists in WT and HET suggest the possibility that increased extracellular retinal DA and altered retinal DA signaling is associated with this DAT variant. Lavoie et al. conducted research on the DAT-knockout mouse model, in which they observed a decrease in rod sensitivity in scotopic condition but no changes in other parameters in both scotopic and photopic conditions [30]. The difference between our findings and theirs may be due to the different photopic protocols we applied. Our protocols emphasize the time-dependent light adaptation of the retina from a dark condition whereas they assessed retinal responses to light stimulus of increasing intensities after light adaptation. However, we did not observe a change in the overall content of retinal DA, DA metabolites, and DA turnover with tissue level measurements in Val559 DAT HOM animals. This result in the retina is consistent with a lack of change in overall DA content in the striatum, cortex, and midbrain previously reported in this mouse model [36]. This finding is also in agreement with DAT-knockout mice, in which no change was found in DA tissue content in the retina [30]. Since our experiments used tissue extracts and measured global DA content, it is possible that DA in the vesicular reservoir, or in the cytosol of synaptic terminals, masked any changes in the extracellular DA in our measurements.

### Val559 DAT blunts retinal responses to AMPH

AMPH alters the action of presynaptic DAT to terminate DA signaling, leading to DA efflux and increased synaptic DA. Previously, we found AMPH evokes DA elevation in WT animals causing increased D2R-mediated IPSC amplitude and duration in striatal brain slices and enhances horizontal and vertical activity at the behavior level. However, in Val559 DAT HOM mice, AMPH-induced DA release and hyperactivity are significantly blunted [36]. Our observations in the retina parallel these behavioral and synaptic observations from the brain. Application of AMPH increased the photopic b-wave amplitude in WT, but failed to do so in the Val559 DAT HOM. The b-wave amplitude in Val559 was not affected by AMPH, indicating a possible ceiling effect due to partial depletion of DA reservoir caused by anomalous DA efflux in Val559 HOM. Although the AMPH is given systematically, the readout of photopic ERG amplitudes represents the changes in the local retinal DA, as supported by findings in retinal dopamine-specific knockout mouse line, where brain DA is normal but retinal DA is depleted [12].

### Sex dependency

In our study, we observed a different effect of the DAT Val559 variant in male and female mice. DAT Val559 failed to increase the photopic b-wave amplitude in females, an effect mainly ascribable to the higher baseline b-wave amplitude of WT females. This may be due to elevated tonic DA signaling compared to WT males. In humans, adult females have significantly different neuro-retinal functions from males, with females exhibiting larger scotopic b-wave amplitude [44], earlier onset of photopic oscillatory potentials [45], and shorter implicit time both locally and globally [46]. In patients with Parkinson’s disease, where retinal dopaminergic signaling and multiple dimensions of visual function are compromised, the reduction of amplitude of visually evoked cortical potentials (VEP) and pattern ERG is significantly different in male and female patients [47]. Estrogen has been suggested to increase DA synthesis, metabolism, and transport [48–51] and protect dopaminergic neurons from neurotoxic damage [52, 53]. ADHD, another psychiatric disorder closely associated with dopaminergic function, exhibits sex differences, with a higher prevalence in men (i.e., ~ 2.1–5.4%) and lower in women (i.e., ~ 1.1–3.2%) [54, 55]. Taken together, these results suggest that the increased penetrance of DAT Val559 on retinal function in males may be due to lower baseline DA signaling in males vs. females.

### ERG as a potential biomarker for ADHD

ADHD has a prevalence rate of 4–12% [25, 56, 57] in school-age children and 4–5% in adults [55, 58, 59], exhibiting symptoms of inattention, hyperactivity, and impulsivity. Current diagnostic methodology relies on behavioral observations and questionnaires without reliance on biomarkers that could help distinguish alternative disorders or subtypes or assist in quantifying treatment response.

Here, we provide evidence that the altered DA signaling induced by a human DAT mutation associated with ADHD can be detected in a mouse model using the non-invasive ERG. Apart from this specific rare DAT Val559 variant, the potential use of ERG and measurements of vision-related responses have broader implications in diagnoses of ADHD caused by changes in extracellular DA particularly mediated by DAT. Previous studies in several other psychiatric disorders, including seasonal affective disorder (SAD) [60, 61] and autism spectrum disorders (ASDs), have shown changes in visual measurements [62–64]. Patients with SAD exhibit decreases in both rod sensitivity and cone-driven b-wave amplitude and a lengthening of cone-driven b-wave implicit time during depression episode. Retinal anomalies represent a state marker of SAD and can be normalized in summertime or by a 4-week bright light therapy treatment [61]. In addition to retinal functions, pupillary light reflex (PLR) measurements are also valuable for diagnosing early autism. A delayed pupil response to light was observed in children with ASDs. Using PLR latency alone, ASD group can be discriminated from the individuals with typical development with a high cross-validated success rate (89.6%) [62]. Additionally, Constable et al. suggested ASD patients have altered cone-ON bipolar signaling. They observed reduced b-wave amplitude across the ASD group under light-adapted conditions, along with the ON response of the prolonged flash ERG. Some ASD individuals also showed subnormal dark-adapted ERG b-wave amplitudes [64].

## Conclusions

In conclusion, DAT Val559 homozygosity increases light-adapted retinal responses consistent with increased DA signaling in the retina. This effect is sex-dependent because of relatively increased photopic ERG amplitudes in WT female mice, which may be due to effects of sex hormones on retinal DA function. In male mice, DAT Val559 also blunts the retinal responses to AMPH, consistent with its known effects on dopaminergic brain circuit function. As this DAT variant is associated with DA-related developmental and psychiatric disorders in humans [34], our findings suggest the possibility of altered retinal and visual function in those harboring the DAT Val559 allele and, more broadly, patients whose ADHD may be supported by ADE triggered through altered DAT regulatory pathways.

## Abbreviations

ADE: Anomalous dopamine efflux
ADHD: Attention-deficit/hyperactivity disorder
AMPH: Amphetamine
ASD: Autism spectrum disorder
BPD: Bipolar disorder
DA: Dopamine
DAT: Dopamine transporter
ERG: Electroretinogram
HET: Mice heterozygous for the dopamine transporter Val559 variant
HOM: Mice homozygous for the dopamine transporter Val559 variant
PLR: Pupillary light reflex
SAD: Seasonal affective disorder
